# Effectiveness of Stroke Early Supported Discharge: Analysis From a National Stroke Registry

**DOI:** 10.1161/CIRCOUTCOMES.119.006395

**Published:** 2020-07-17

**Authors:** Rebecca J. Fisher, Adrian Byrne, Niki Chouliara, Sarah Lewis, Lizz Paley, Alex Hoffman, Anthony Rudd, Thompson Robinson, Peter Langhorne, Marion F. Walker

**Affiliations:** 1 University of Nottingham, United Kingdom (R.J.F., A.B., N.C., S.L., M.F.W.).; 2 King’s College London, United Kingdom (L.P., A.H., A.R.).; 3 University of Leicester, United Kingdom (T.R.).; 4 University of Glasgow, United Kingdom (P.L.).

**Keywords:** consensus, England, hospitals, registries, survivors

## Abstract

**Background:**

Implementation of stroke early supported discharge (ESD) services has been recommended in many countries’ clinical guidelines, based on clinical trial evidence. This is the first observational study to investigate the effectiveness of ESD service models operating in real-world conditions, at scale.

**Methods and Results:**

Using historical prospective data from the United Kingdom Sentinel Stroke National Audit Programme (January 1, 2016–December 31, 2016), measures of ESD effectiveness were “days to ESD” (number of days from hospital discharge to first ESD contact; n=6222), “rehabilitation intensity” (total number of treatment days/total days with ESD; n=5891), and stroke survivor outcome (modified Rankin scale at ESD discharge; n=6222). ESD service models (derived from Sentinel Stroke National Audit Programme postacute organizational audit data) were categorized with a 17-item score, reflecting adoption of ESD consensus core components (evidence-based criteria). Multilevel modeling analysis was undertaken as patients were clustered within ESD teams across the Midlands, East, and North of England (n=31). A variety of ESD service models had been adopted, as reflected by variability in the ESD consensus score. Controlling for patient characteristics and Sentinel Stroke National Audit Programme hospital score, a 1-unit increase in ESD consensus score was significantly associated with a more responsive ESD service (reduced odds of patient being seen after ≥1 day of 29% [95% CI, 1%–49%] and increased treatment intensity by 2% [95% CI, 0.3%–4%]). There was no association with stroke survivor outcome measured by the modified Rankin Scale.

**Conclusions:**

This study has shown that adopting defined core components of ESD is associated with providing a more responsive and intensive ESD service. This shows that adherence to evidence-based criteria is likely to result in a more effective ESD service as defined by process measures.

**Registration:**

URL: http://www.isrctn.com/; Unique identifier: ISRCTN15568163.

## Abstract 2

Supplemental Digital Content is available in the text.

**What Is Known**

- Stroke care guidelines worldwide recommend the provision of early supported discharge.
- Implementation of early supported discharge in real-world settings has been highly variable.

**What the Study Adds**

- The study supports the use of an early supported discharge consensus score to quantify adoption of evidence-based core components of early supported discharge.
- Extension of a national stroke registry can offer important opportunities to evaluate community stroke service delivery.

Stroke is one of the main causes of adult disability and there is strong research evidence that provision of stroke specialist rehabilitation enhances recovery.^1^ In England, the recent National Health Service (NHS) Long Term Plan has made renewed recommendations for implementation of care models for stroke rehabilitation in practice with increased investment in community healthcare services.^2^ Stroke early supported discharge (ESD) is a multidisciplinary team intervention that facilitates discharge from hospital and delivery of stroke specialist rehabilitation at home.^3^ Based on cumulative evidence from clinical trials, stroke care guidelines in England and worldwide recommend the provision of ESD as part of an evidence-based stroke care pathway.^4–9^

Implementation of ESD in real-world settings, however, has been highly variable. In the United Kingdom, ESD services differ across the country, and, in some regions, ESD is still not offered at all.^10^ In most other high-income countries, ESD has not been well developed in practice, resulting in some locally established services, but a lack of large scale implementation.^11^ In fact, in many countries, it is unclear what provision of rehabilitation there is for stroke survivors beyond the hospital setting, with the majority of national audits or registries focusing on acute stroke care.^12^

It is, therefore, not known what models of ESD are in operation in practice (if at all), or how close they are to the evidence-based models with demonstrated effectiveness in clinical trials. There are also unanswered questions relating to implementation of ESD in rural settings, with the original clinical trials mainly conducted in urban sites.^3,13^ It remains unclear whether benefits of the ESD intervention are achieved when services are implemented at a large scale, in the real world.

The study focuses on provision of care at a particularly distressing time: when stroke survivors leave hospital and face the consequences of stroke back at home. Clinical guidelines recommend that ESD services should provide responsive and intensive rehabilitation (with treatment at home beginning within 24 hours of hospital discharge) with the aim to promote stroke survivor recovery.^4–9^ By investigating if and how these aspects of an effective ESD service can be realized in practice, this study aims to inform provision of evidence-based care for stroke survivors.

The Sentinel Stroke National Audit programme (SSNAP) is the national stroke register of England, Wales, and Northern Ireland in which all acute admitting hospitals and postacute stroke teams are mandated to participate.^14^ SSNAP has played a key role in monitoring performance and improving provision of acute stroke care. Collection of SSNAP data from community stroke services now offers a unique opportunity to investigate the large scale impact of ESD.

Our previous research has hypothesized that the active ingredients of ESD can be defined with evidence-based core components^13^ and that these core components are essential characteristics that need to be implemented for the ESD intervention to be effective in practice.^15^ The aim of the current study was to determine if such core components had been adopted by ESD teams in real-world settings in England and whether these related to realized benefits of ESD.

## Methods

### Study Design

We present results from an observational cohort study (Figure), conducted as part of an overall mixed-method study.^16^ The study protocol was approved by a University of Nottingham Ethics Committee and the United Kingdom Healthcare Quality Improvement Partnership Data Access Request Group. Data access requests should be directed to the Healthcare Quality Improvement Partnership as the joint data controller and SSNAP as the data provider. The study protocol including statistical analysis plan is available online.^16^ We determined a priori, a sample size of 4750 patients for a study power of 80% to detect standardized effect sizes of 0.25 for each outcome.

**Figure.**
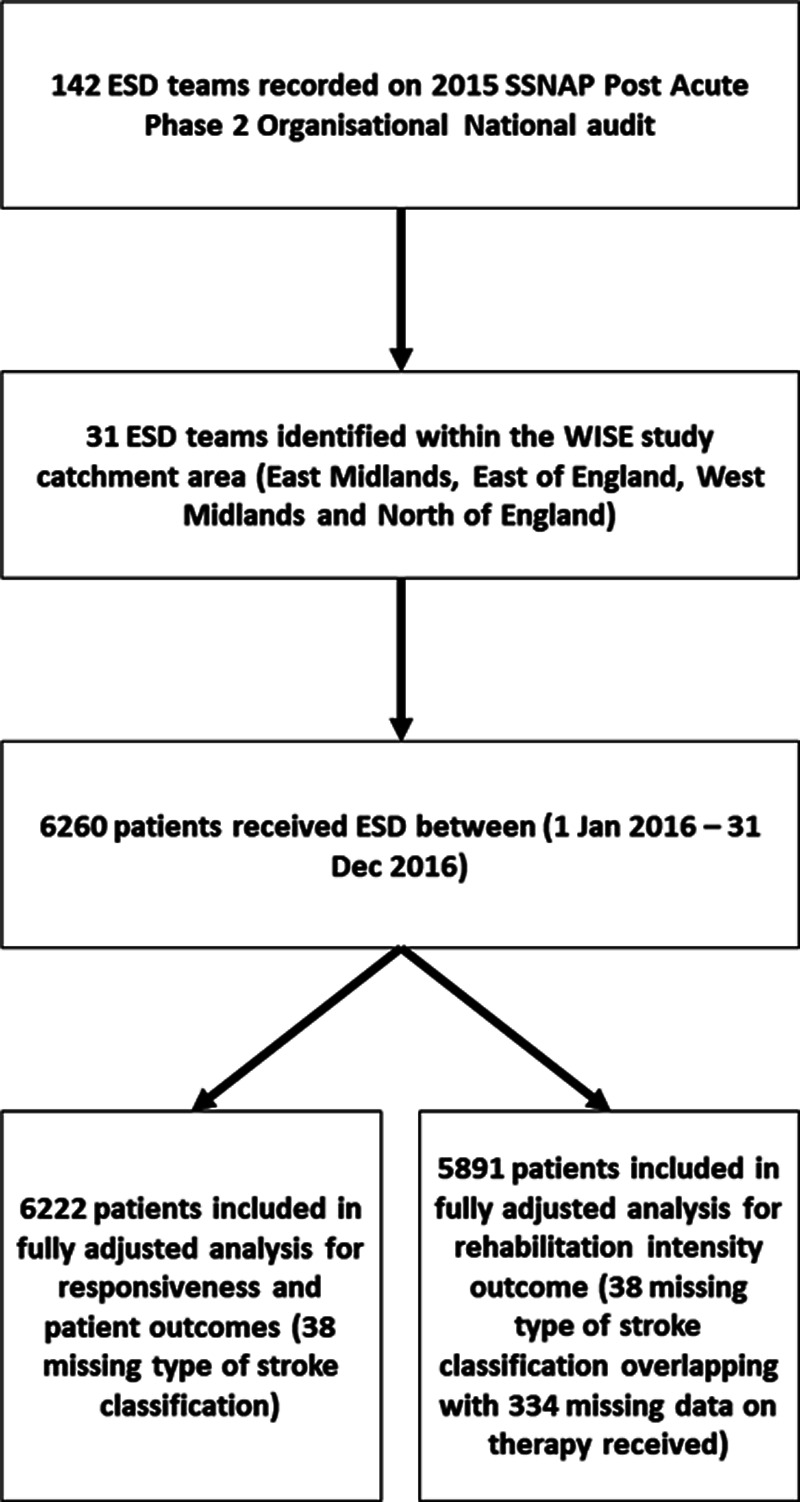
**Study design flow chart.** ESD indicates early supported discharge; SSNAP, Sentinel Stroke National Audit Programme; and WISE, What is the Impact of Stroke Early supported discharge.

### Setting

ESD services were sampled across a large geographic area of England. The sampling strategy was devised in accordance with the overall mixed-method study design and included all ESD services in specific regions of England.^16^ Here, we report findings from quantitative investigation of ESD effectiveness across West and East Midlands and East of England (across which a specific initiative to promote ESD was initiated in 2010) and the North of England, a region with a defined lack of ESD.^10,17^

### Data Sources and Participants

The aim of the study was to examine the association between ESD service models, and process, and patient outcome measures of ESD effectiveness. Information about ESD service models included in the study was obtained from SSNAP postacute organizational audit data, which was published freely in the public domain in 2015.^10^ ESD teams had participated in the 2015 postacute organizational audit by completing questionnaires investigating organizational characteristics of their service in relation to evidence-based standards, distributed and collated by SSNAP.^10^

Patient-level SSNAP data are entered by clinical teams onto a secure webtool with real-time data validations to ensure data quality.^14^ Historical prospective clinical (patient level) SSNAP data from all SSNAP participating ESD teams in the geographic area of interest (n=31) were obtained with permission from the Healthcare Quality Improvement Partnership.

### Key Predictor—ESD Consensus Score

We hypothesized that adoption of evidence-based core components of ESD was important for the ESD intervention to be effective in practice. An ESD consensus score was developed using defined evidence-based core components of ESD as outlined in an international consensus document and evidence-based postacute organizational audit criteria utilized by SSNAP in the postacute audit (Table 1).^10,13^ Statements defining core components of ESD from the consensus document (derived using an international panel and modified Delphi process) were compared with items from the postacute organizational audit questionnaire used previously by SSNAP. Using this process, a 17-item ESD consensus score was designed by the study team to measure adoption of core components of an ESD service model, for example, team composition (core team and others), staff training, team meetings, and service specificity (Table 1). This 17-item ESD consensus scoring system was then applied to organizational audit questionnaire data (categorical data previously collected by SSNAP) for each of the 31 ESD teams involved in the study.

**Table 1.**
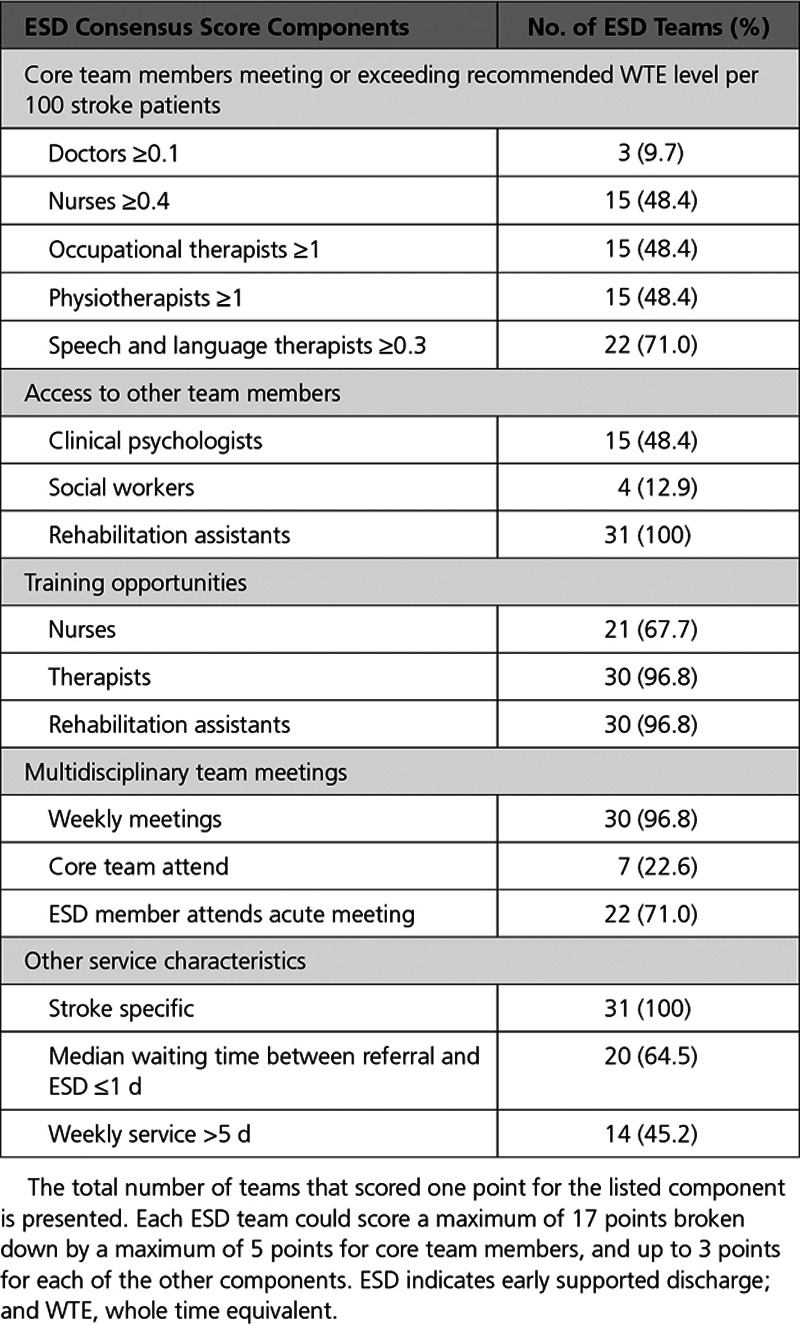
ESD Consensus Score Components Across 31 ESD Teams

Based on the proposed scoring system, an ESD team could score a maximum of 17 points broken down by a maximum of 5 points for core team members and up to 3 points for each of access to other team members, training opportunities, multidisciplinary team meetings, and level of service provided (Table 1). Adoption of evidence-based core components was measured by calculating an ESD consensus score for each of the 31 teams.

### Process and Patient Outcome Measures

Measures of effectiveness of ESD were based on clinical guidelines and ESD systematic review recommendations and were dependent on what patient-level SSNAP data variables were collected routinely.^3,4^ Using historical prospective SSNAP clinical data (January 1, 2016–December 31, 2016), measures of ESD effectiveness were “days to ESD” (number of days from hospital discharge to first face-to-face contact; number of patients=6222), “rehabilitation intensity” (total number of treatment days/total days with ESD; number of patients=5891), and stroke survivor outcome (modified Rankin Scale at discharge from ESD; number of patients=6222). The measure of rehabilitation intensity was based on established approaches used by SSNAP.^18^ The modified Rankin Scale score, routinely collected at discharge from the ESD service, was used as the stroke survivor outcome and in analysis was controlled for by modified Rankin Scale at discharge from hospital.

“Days to ESD” was a binary variable (0=ESD team sees the patient within 1 day; 1=ESD team sees patient after 1 day or more). “Rehabilitation intensity” was a natural log-transformed continuous measure (the results presented in the text have been back-transformed to give the percent change per unit). The stroke survivor outcome measure of modified Rankin Scale (at ESD discharge) was treated as an ordinal categorical variable with the following categories of increasing dependency: 0, 1, 2, 3, 4 to 5 (combined due to low patient numbers).

### Other Variables

To investigate the effect of ESD consensus score on process and patient outcomes, we controlled for many covariates, which were measured at the ESD team level (level 2 in our multivariate model described below), or patient level (level 1).

We identified a need to control for the effect of preceding hospital care and geographic context of delivery of rehabilitation. At the site (or ESD team) level, we included 2 confounding variables: a rurality score and a hospital SSNAP rating score. The rurality score was based on the Rural-Urban classification reported for the geographic area associated with the NHS clinical commissioning group who had procured each ESD team.^19^ Each commissioning group in England has a geographic area over which it operates to procure NHS services. Where an ESD team included in this study was managed by multiple commissioning groups, then the weighted average level of rurality was calculated, based on the prevalence of stroke and transient ischemic attack in that commissioning area (figures obtained from NHS Quality and Outcomes Framework).^20^

The hospital rating scores used in this study were an overall quality rating for each hospital obtained from SSNAP (total key indicator score derived across ten domains of stroke care with adjustments made for case ascertainment levels and the quality of data submitted to SSNAP). The score for each referring hospital (associated with each ESD team of interest) was used as an indication of the overall standard of inpatient care before ESD referral.^21^ For ESD teams with multiple discharging hospitals, a weighted average SSNAP rating score was calculated based on the number of patients being discharged to those ESD teams.

To account for differing patient characteristics between ESD teams, we also included variables at the patient level. These were patients with stroke characteristics, reflecting validated stroke case-mix models and collected as part of the SSNAP data set and included age, sex, prestroke independence, comorbidities, National Institutes of Health Stroke Scale score on admission, type of stroke, and modified Rankin score at discharge from hospital.^22,23^

### Statistical Analyses

Multilevel modeling was used to investigate relationships between ESD model and process and patient outcomes in an approach consistent with previous observational studies of this type.^22–25^ Combining SSNAP postacute organizational audit data at the site (ESD team) level with SSNAP clinical audit data at the patient level, we fitted generalized linear mixed models on 2 levels, ESD team (level 2), and patient nested within ESD team (level 1) to process and patient outcomes. Covariate adjustments were made for site (ESD team; level 2) and patient (level 1) variables. Models were fitted for “days to ESD,” “rehabilitation intensity,” and modified Rankin Scale score at ESD discharge using multilevel logistic, linear, and ordinal logistic models, respectively.

The ESD consensus score was used in 3 different ways: total score, disaggregated by component, and where appropriate, as an individual item. We began by assessing the significance of the total score in relation to our outcomes of interest (both unadjusted and adjusted). If a significant association was found then further analyses by components and then individual items were conducted to uncover the key driver(s) behind the significant association(s). Any statistically significant components were tested for linearity (using likelihood ratio tests) to assist with substantive inference. Where possible variables were interpreted in a continuous fashion otherwise they were treated as categorical if any variable could not be interpreted in a linear way.

We chose multilevel modeling to evaluate the effectiveness of ESD service provision as it can accommodate and appreciate the variation that may exist within and between different ESD teams. Furthermore, the intraclass correlation coefficient was calculated as a measure of proportion of the total variance in outcomes, which is attributable to variance within ESD services as opposed to between services.

The adequacy of different statistical models was compared using the log-likelihood, Akaike Information Criterion, and Bayesian Information Criterion values from single level and multilevel regression models for each outcome variable with multilevel preferable on each occasion. Multicollinearity was investigated by examining variance inflation factor scores of all predictor variable sets and was found not to be an issue. Covariate linearity was examined by checking the consistency of a linear trend in relation to each outcome variable. To explore the impact of missing data, we conducted sensitivity analysis excluding any teams that had missing outcome data; no substantial differences were found.

A 2-tailed significance level of 0.05 was used in all hypothesis tests. We performed all analyses using Stata/SE 15.1 (Statacorp).

## Results

Total ESD consensus scores across the 31 teams varied between 5 and 15 (mean [SD], 10.6 [2.4]) with no team achieving 100% adherence, reflecting that a range of ESD models had been adopted (see Table 1). For the range of ESD models, there was a mixture of urban and rural settings (mean level of rurality [SD]: 35.6 [21.8]), as well as varying performance of associated referring hospitals (mean SSNAP hospital rating score [SD]: 72.2 [12.1]).

Data from 6260 patients with a completed National Institutes of Health Stroke Scale score were included in the primary analysis and their characteristics are shown in Table 2. The majority, 91.9%, of patients had a mild or moderate stroke (National Institutes of Health Stroke Scale <15). The most common age group was 70 to 79 years (30.8%), and 4151 (66.3%) of patients were functionally independent before their stroke (modified Rankin Scale=0).

**Table 2.**
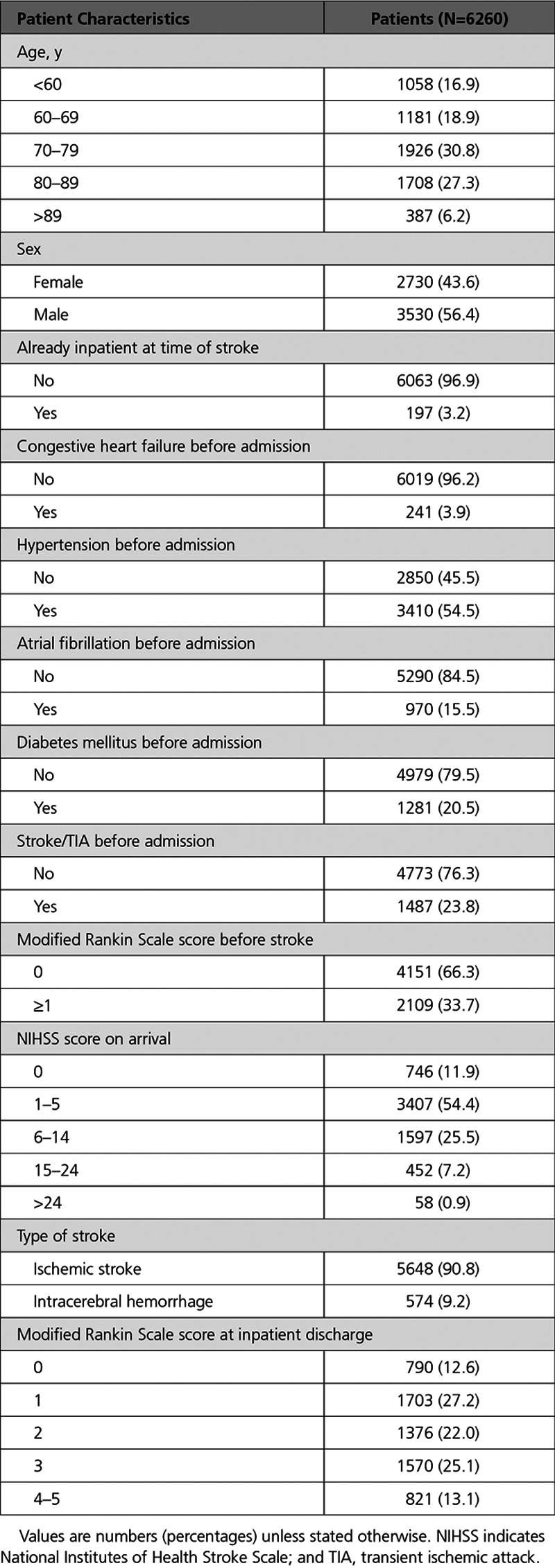
Patient-Level Variables

In terms of the outcomes, 69% of sampled patients were seen after ≥1 day with 31% seen within 1 day for the “days to ESD” variable. The median rehabilitation intensity value of sampled patients was 0.38 treatment days for every day with the ESD team with the 25th percentile being 0.19 and the 75th percentile being 0.59. For the stroke survivor outcome measure, 9% of sampled patients were classified as moderate to severe at ESD discharge (modified Rankin scale score, 4–5) with percentages of patients with a modified Rankin scale of 0, 1, 2, 3 as 9%, 31%, 31%, 20%, respectively.

Results of the multilevel modeling are presented in Tables 3 through 5. The degree of clustering was greater for process measures “days to ESD” and “rehabilitation intensity” compared with the patient outcome measure of modified Rankin Scale (adjusted intraclass correlation coefficients respectively, 0.56, 0.26, 0.08).

**Table 3.**
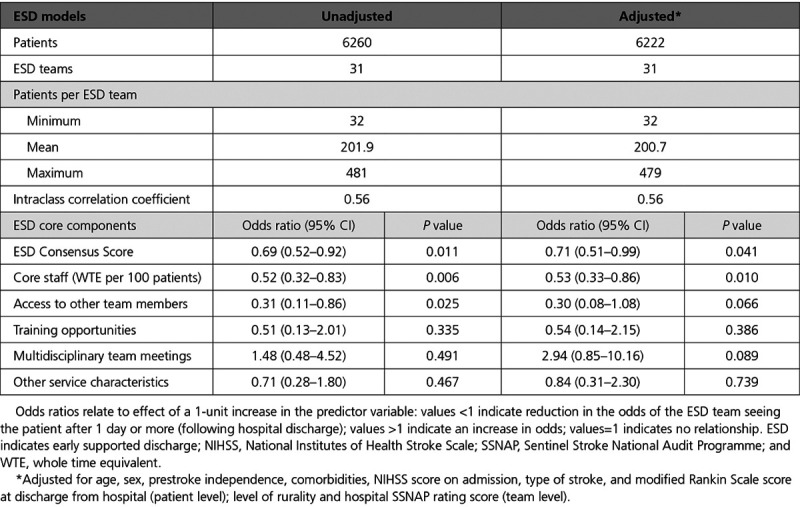
Association Between ESD Consensus Score/Components and Days to ESD

Results for the association between total ESD consensus score and the “days to ESD” variable are shown in Table 3, unadjusted and adjusted for all patient characteristics, level of rurality, and weighted average SSNAP hospital score. Odds ratios are presented in Table 3 with percentage odds reported here. From the adjusted results, a 1-unit increase in the ESD score was associated with an odds ratio of 0.71 (95% CI, 0.51–0.99), or in other words, with a reduced odds (by 29%) of the ESD team seeing the patient after ≥1 day following hospital discharge. Hence an increase in ESD consensus score was associated with a more responsive ESD service. Exploring the effect of components, this association appeared to be driven by having more core team members meeting or exceeding recommended whole time equivalent level per 100 patients with stroke (a 1-unit increase was significantly associated with a 47% reduction in the odds of the ESD team seeing the patient after ≥1 day [95% CI, 14%–67%]). There was some evidence, at borderline significance, of an effect of access to other team members (reduced odds of 70% [95% CI, −8% to 92%]). Further investigation at an individual item level showed that having access to a social worker was associated with more responsive ESD service with 97% reduced odds of the ESD team seeing the patient after ≥1 day (95% CI, 61% to 99%).

Table 4 presents the linear multilevel model results for the rehabilitation intensity outcome measure, unadjusted and adjusted for all patient characteristics, weighted level of rurality, average SSNAP hospital score, and total ESD consensus score. Focusing on the adjusted results and coefficients (presented as percentages here), the ESD consensus score was significantly associated with treatment intensity such that a 1-unit increase in ESD consensus score increased treatment intensity (total number of treatment days / total days with ESD) by 2% (95% CI, 0.3%–4%). With respect to this significant association, holding weekly multidisciplinary team meetings with the core team attending (Table 1) and a member of the ESD team attending the acute meetings were all positively associated with increased rehabilitation intensity; specifically an average 8% (95% CI, 0.9%–16%) improvement in rehabilitation intensity.

**Table 4.**
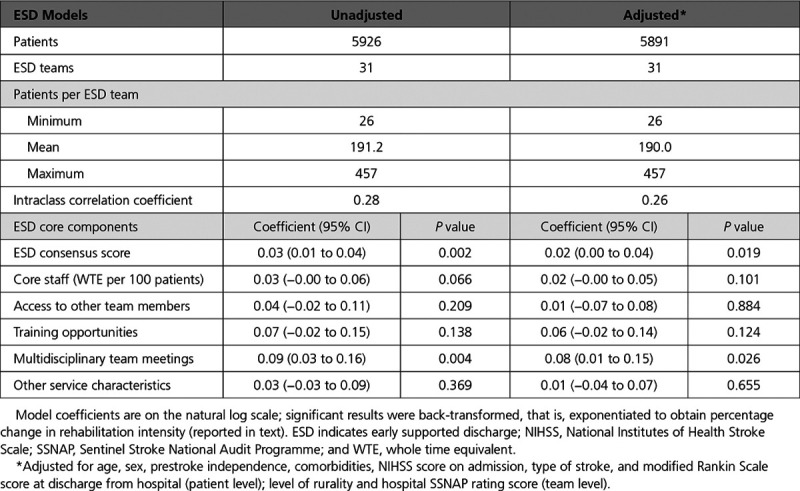
Association Between ESD Consensus Score/Components and Rehabilitation Intensity

Table 5 presents the ordinal logistic multilevel model results for the patient outcome measure, unadjusted and adjusted for all patient characteristics, weighted average SSNAP hospital score, and level of rurality. There was no significant association between ESD consensus score and the stroke survivor outcome measured by the modified Rankin Scale at ESD discharge.

**Table 5.**
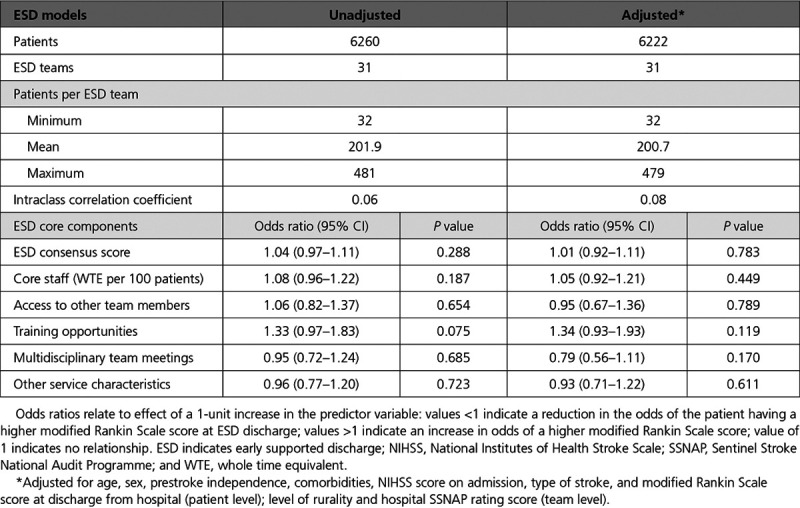
Association Between ESD Consensus Score/Components and Stroke Survivor Outcome

Site-level control variables, namely percentage rurality and hospital SSNAP rating score, had no statistically significant relationship with any of the outcomes.

## Discussion

This study was designed to inform large scale implementation of ESD by evaluating its effectiveness in real-world conditions, at scale, using recommended methodology.^24^ This addresses recent recommendations for investment in stroke rehabilitation made in NHS England’s Long Term Plan and lack of large scale development of ESD worldwide.^2,11^ The study found that a variety of ESD service models have been adopted in regions of interest, as reflected by variability in the ESD consensus score. Controlling for patient characteristics and other confounding variables, ESD consensus score was significantly associated with a more responsive ESD service (reduced odds of patient being seen after ≥1 day) and increased rehabilitation intensity, but no effect on stroke survivor outcome as measured by the modified Rankin scale was demonstrated. We conclude that adopting defined core components of ESD was associated with providing a more responsive and intensive ESD service, suggesting that adherence to evidence-based criteria is likely to result in more effective services in practice. This builds on methods used to investigate the organization of stroke unit care bringing a much-needed focus on community-based stroke care.^25^

There are limitations inherent to observational data, which we aimed to address with the study design. Although the study used a large sample of stroke patient data, it must be acknowledged that data from a relatively small sample of ESD services were used in this study; further research would be required to confirm wider transferability, particularly beyond England. A key feature of this study was development of the ESD consensus score. Although we acknowledge that more in depth investigation of ESD model features is required to make definitive conclusions, this approach offered a useful way to quantify adoption of core components for quantitative analytical purposes.^16^ It provided a simple means by which to evaluate services based on international consensus and clinical guidelines relating to ESD.^4,5,13^ We attempted to control for several confounders; however, we cannot rule out the possible influence of unobserved variables. Outcomes of interest were reliant on a relatively small SSNAP dataset, entered by community stroke service staff. Findings are reliant on accurate reporting, and the possibility of bias cannot be excluded. It should also be noted that previous studies have suggested ESD reduces length of hospital stay; investigation of this, using hospital SSNAP data, will be reported in a further paper.^16^

Clinical guidelines emphasize the importance of seamless transfers of care and previous studies have reported the negative impact of delayed or uncoordinated transfers on patients.^26,27^ In addition to teams with higher total ESD consensus scores being more likely to see patients sooner, findings highlighted the importance of the ratio of staff to patients. Hence, teams that met (or exceeded) consensus recommended whole time equivalent levels of staff per 100 patients with stroke were more likely to be responsive, emphasizing the need for ESD services to be appropriately resourced.^13^ Previous studies have also highlighted transfer problems relating to lack of joint working between health and social care.^26–28^ This study adds to this debate by highlighting the importance of access to a social worker as part of the ESD team.

ESD has been recommended as a high-intensity rehabilitation intervention, with guidelines and systematic reviews referring to daily or 4 to 5 visits per week.^3–5^ In this study, intensity of rehabilitation delivery was measured by calculating the percentage of treatment days in relation to the patients’ total time with the ESD service. In addition to total ESD score, the multidisciplinary team working component was associated with increased intensity of rehabilitation delivery. This resonates with previous studies emphasizing the importance of multidisciplinary team working in delivery of stroke care and in particular multidisciplinary team meetings.^29–31^

Routine collection of patient outcomes in SSNAP is currently limited to use of the modified Rankin scale. Findings could be interpreted such that the model of ESD adopted did not influence patient outcomes as measured by the modified Rankin scale; however, caution is required. Robust modified Rankin scale data were only available at discharge from the ESD service (as opposed to at a later follow-up stage), and so it is possible that there was not sufficient time to investigate ESD effects. There was also a lack of variability of this outcome measure in the study, possibly reflecting a focus of ESD services on treatment of mild to moderate stroke survivors. There have also been concerns from teams themselves about reliability of use of this score across the stroke care pathway.^32^ We suggest routine collection of additional validated patient outcome measures (eg, measuring activities of daily living, general health/mood, and quality of life) at longer follow-up periods in national stroke audits or registries is required.^3,13,15^

Finally, at site level, the lack of effect of rurality was surprising. It is encouraging that we found examples of evidence-based ESD models in rural regions, yet reported challenges with healthcare provision in these settings cannot be overlooked.^33,34^ Further investigation of the impact of geographic location on implementation of ESD is required.

## Conclusions

Original clinical trials of ESD were conducted across the world, and implementation of ESD is recommended in many countries’ stroke guidelines.^4–9^ This study supports the use of an international ESD consensus document as a means to guide implementation of effective, evidence-based ESD in practice.^13^ We suggest extension of national stroke registries with inclusion of community stroke data would offer important opportunities to evaluate stroke service delivery beyond the hospital setting.^12^ This would go some way towards addressing current gaps in provision of stroke rehabilitation which exist globally, moving towards the goal of ensuring stroke survivors receive the evidence-based care they deserve.

## Acknowledgments

Fisher (Principal Investigator), Byrne, Lewis contributed to study design, data analysis and interpretation, and article writing; Chouliara, Walker, Robinson, Langhorne contributed to study design and article writing; Paley, Hoffman, Rudd are Sentinel Stroke National Audit Programme (SSNAP) collaborators. We thank the many people and organizations participating in SSNAP and members of the SSNAP collaboration.

## Sources of Funding

This research was funded by the National Institute for Health Research (NIHR), Health Services and Delivery Research (HS&DR) Programme (16/01/17). Dr Fisher is funded by the Stroke Association (TSA LECT 2016/01 Stroke Association HRH the Princess Margaret Senior Lecturer Award). The Sentinel Stroke National Audit Programme is commissioned by the Healthcare Quality Improvement Partnership and funded by National Health Service (NHS) England and the Welsh Government. The views expressed are those of the authors and not necessarily those of the National Health Service (NHS), the National Institute for Health Research (NIHR), or the Department of Health and Social Care.

## Disclosure

None.

## Supplementary Material


